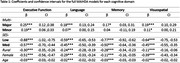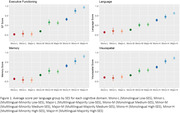# Association of Majority versus Minority First Language Multilingualism and Socioeconomic Status on Cognition among Older Indian Adults

**DOI:** 10.1002/alz.090676

**Published:** 2025-01-03

**Authors:** Iris M. Strangmann, Justina F Avila, Sarah Petrosyan, Erik Meijer, Emma Nichols, Shrikanth Narayanan, Leon M. Aksman, Jinkook Lee, Miguel Arce Rentería

**Affiliations:** ^1^ Columbia University, New York, NY USA; ^2^ Columbia University Irving Medical Center, New York, NY USA; ^3^ University of Southern California, Los Angeles, CA USA; ^4^ Mark and Mary Stevens Neuroimaging and Informatics Institute, University of Southern California, Los Angeles, CA USA

## Abstract

**Background:**

Some research suggests that multilingualism confers a cognitive advantage, but this association may be confounded by linguistic and socioeconomic factors. Multilinguals can differ on their first language such that it could be a societal majority‐ or minority‐language, resulting in distinct reasons for‐ and experiences with‐ becoming/being multilingual, along with different socioeconomic opportunities. We evaluated the association of multilingualism (Monolingual, Multilingual‐Majority, Multilingual‐Minority) and socioeconomic status (SES) on cognition among older adults.

**Method:**

The analytic sample included 3,918 older adults (65% Monolingual, 23% Multilingual‐Majority, and 12% Multilingual‐Minority) from the Longitudinal Aging Study in India – Diagnostic Assessment of Dementia (LASI‐DAD). Participants reported their first and (any) additional language(s), and completed a cognitive battery assessing executive function, language, memory, and visuospatial domains. Majority language within residential state was determined per the Indian Census. SES was defined by years of education, caste, consumption, and parental education. SES scores were derived through factor analysis and grouped into tertiles (High‐, Medium‐, Low‐SES). The associations of multilingualism and SES were evaluated using Multilevel Analysis of Individual Heterogeneity and Discriminatory Accuracy (MAIHDA), adjusting for age, sex/gender, and rurality.

**Result:**

Multilingual‐majority status was associated with better cognitive performance across all domains compared to monolinguals, while multilingual‐minority status performed better on all domains except memory (Table 1). Relative to high‐SES, lower‐ and medium‐SES were associated with worse performance across all domains. Results suggest an interaction between language group and SES (Figure 1). While high‐SES monolinguals performed worse than their multilingual counterparts in all domains ‐ except for memory when compared to high‐SES multilingual‐minority participants ‐ they outperformed low‐ and medium‐SES multilinguals. Although high‐SES multilinguals outperformed low‐/medium‐SES multilinguals in all domains, there were no differences between majority/minority‐multilinguals of equal SES.

**Conclusion:**

Both multilingualism, regardless of majority‐minority first language, and socioeconomic status were associated with cognitive benefits. While a multilingual cognitive advantage was more apparent among those with high SES, this advantage did not surpass the independent benefit of high SES, since monolinguals with high‐SES outperformed multilingual older adults with low‐ and medium‐SES. These results suggest that the potential benefits of multilingualism on cognition differs across life‐course socioeconomic determinants of health.